# How to Suppress Mineral Loss and Stimulate Anabolism in Postmenopausal Bones with Appropriate Timing of Exercise and Nutrients

**DOI:** 10.3390/nu16060759

**Published:** 2024-03-07

**Authors:** Katarina T. Borer

**Affiliations:** School of Kinesiology, The University of Michigan, Ann Arbor, MI 48104, USA; katarina@umich.edu; Tel.: +1-(734)-249-8809

**Keywords:** bone osteogenesis, postmenopausal bone, nutritional requirement, circadian timing, meal and exercise timing, rest-inserted exercise

## Abstract

**Background**. Bone Health and Osteoporosis Foundation (BHOF) reports that as of 2023, approximately 10 million of older Americans have osteoporosis and another 44 million have low bone density. Osteoporosis is a serious handicap for the elderly and, in particular, for estrogen-deficient postmenopausal women, as it increases the risk of debilitating bone weakness and fractures. The BHOF recommendations for prevention of osteopenia, osteoporosis and bone fractures are to perform weight-bearing and muscle-strengthening exercises and to take recommended amounts of daily calcium and vitamin D. **Methods**. The purpose of this review is to describe and discuss recent evidence-based research on how to effectively utilize timing of exercise and calorie intake for stimulation of postmenopausal bone anabolism, and to provide this new information in the form of specific and actionable recommendations. **Results**. The five evidence-based recommendations are as follows: 1. Select an appropriate circadian time of day for exercise; 2. Increase walking speed to raise the movement momentum; 3. Eat a weight-maintenance meal one or two hours before the exercise bout; 4. Sustain the duration of walking activity (impulse) for 40 to 45 min; and 5. Repeat effective exercise stimulus 7 to 8 h after the first one to double the anabolic effect. Osteogenesis can also be increased with subthreshold mechanical loading, where needed, under several special circumstances. **Conclusions**. This review should provide pragmatic actionable pointers on how to utilize the idiosyncratic bone responsiveness to timing of movement and meals to prevent osteoporosis and encourage research toward a better understanding of how bone detects adequacy of a mechanical stimulus and determines duration of necessary rest to recover its sensitivity to mechanical stimulation and nutrients.

## 1. Introduction

Bone Health and Osteoporosis Foundation (BHOF) reports that currently approximately 10 million Americans have osteoporosis and another 44 million have osteopenia or low bone density [1]. Osteoporosis is responsible for an estimated two million bone fractures per year in individuals over age 50, where it afflicts 50% of women and 20% of men. Two main recommendations by BHOF for improving bone health and preventing osteoporosis and bone fractures are to perform weight-bearing and muscle-strengthening exercises and to consume daily the recommended amounts of calcium and vitamin D. These recommendations are correct but very general and lack sufficient detail to be specifically useful and actionable. The purpose of this review is to provide additional recent evidence-based details on the importance of timing of movement and meals for suppression of mineral loss and for stimulation of anabolic responses in postmenopausal bone. 

Key mechanical functions of the skeleton are to support the body mass and to allow movement by having bones serve as rigid levers powered by muscles across the articulations of joints. Both functions efficiently and continuously adapt bone structure through bone remodeling to gravitational forces and dynamic rather than static mechanical loading [2,3]. Cells involved in bone remodeling include osteocytes embedded within the rigid hydroxyapatite mineral framework. They sense mechanical strain and activate interaction between osteoclasts that are bone resorbing cells, and osteoblasts that add new bone mineral [4]. This ongoing adaptation of bone structure to changing mechanical loading is seen in the losses of bone mineral density (BMD) and content (BMC) after immobilization of a single limb, after prolonged bed rest, or exposure to zero gravity of outer space [5], a process that results in disuse osteoporosis [6]. On the other hand, dynamic bone loading during movement slows down bone turnover, helps protect bone integrity, and, under appropriate conditions, leads to bone mineral accrual. 

**Figure 1:**
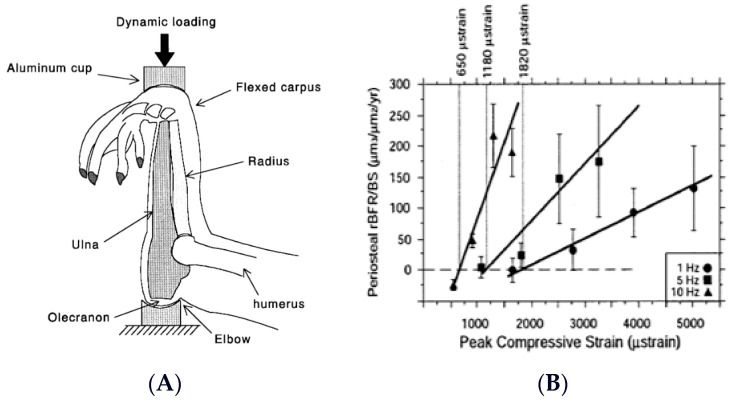
Apparatus for mechanical loading of individual animal long bones (**A**), and demonstration of the capacity of bones to respond both to a range of mechanical loads and to variations in loading frequency (**B**). Modified after [7], Reproduced with the permission of the publisher.

Animal studies performed at the turn of the century used automated apparatus for the mechanical loading of rodent and bird long bones (Figure 1A). They demonstrated not only the importance of mechanical stimulation for bone anabolism, but also responsiveness of bone growth both to large-amplitude, low-frequency loading, characteristic of joint-reaction forces in limb movements and ground reaction forces in locomotion, and to low-amplitude, high-frequency vibrations [7] (Figure 1B). 

**Figure 2:**
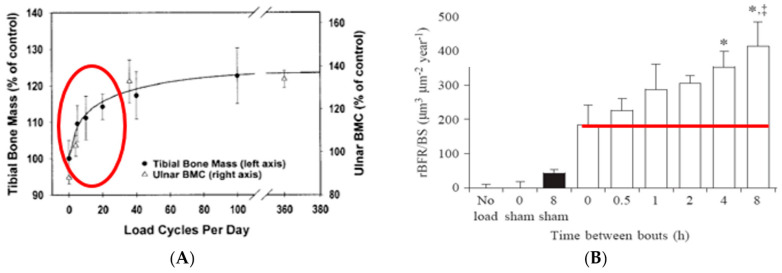
Rapid saturation of the bone anabolic response to mechanical loading of (**A**) a bird ulna [3,9] or of tibias of rats trained to jump onto a platform visible within the red oval [10]. (**B**) The course of an 8-h period of recovery from mechano-refractoriness following 36 cycles of mechanical loading demonstrated by bone growth above the red line. * *p* < 0.05, *, ‡ *p* < 0.01. Modified after [3,9,10,11], Reproduced with the permission of the publisher.

These early studies also demonstrated that bones exhibit idiosyncratic responsiveness to timing of mechanical stimulation. Instead of responding to exercise duration and intensity in a dose-dependent manner with increased mass and strength, as is the case in muscle hypertrophy [8], bones rapidly habituate to an effective mechanical stimulus and cease responding to additional mechanical stimulation for about 6 to 8 h [3,9,10] (Figure 2A). After about 6 to 8 h of this mechano-refractoriness, bone mechanosensitivity returns, and, thus, two effective exercise stimuli separated by a 6- to 8-h refractory period can double the usual anabolic effect [11] (Figure 2B).

In 2003, Turner and Robling used these animal studies as a prototype to offer guidance to humans for exercise strategies designed to increase bone strength [12]. Their recommendations currently face four limitations that subsequent research studies have removed, a technical one, and three conceptual ones. The technical obstacle was the prevalent early method of measuring human bone anabolism with changes in BMD. This drawback was resolved a decade later when circulating markers of bone formation and resorption became widely used as a more rapid measure of bone growth or resorption [13]. Bone markers allowed rapid tracking of human anabolic bone responses through concentration changes in CICP (a carboxyterminal propeptide of type 1 collagen) or P1NP (type1 N-terminal procollagen propeptide) and in bone resorptive responses through changes in CTX (carboxyterminal peptide or C-terminal cross-linking telopeptide of type 1 collagen). Additional frequently used markers of bone metabolism that participate in bone mineralization are the hormone osteocalcin (OC) [14]) and the enzyme bone-specific alkaline phosphatase (BALP) [15]. 

The three conceptual weaknesses were an apparent lack of interest in the role of nutrient calories in modulating exercise effects on bone metabolism, lack of understanding the relevance of circadian timing of exercise, and lack of evidence that the exercise strategies obtained with animal studies could be applied to humans. The narrow focus in BHOF dietary recommendations on the importance of vitamin D and calcium was justified by the key role of bone in plasma calcium homeostasis [16]. The skeleton is the largest repository of calcium in the body (1–2% of body mass) and plays a central role in regulating plasma calcium concentration. A decline in plasma calcium stimulates a sustained increase in parathyroid hormone (PTH) secretion, which then corrects the circulating calcium deficit by activating bone mineral resorption. However, several lines of evidence for the skeletal role in energy balance, and dependence of bone metabolism on nutrition, were seldom considered in the context of bone responses to mechanical loading. The skeleton provides emergency nutrient energy during starvation by breakdown of protein collagen, in addition to releasing 16.5 g of bone mineral for each kilogram of body fat lost [17]. There is a regular daily cycle of nocturnal bone mineral loss that is corrected during daytime feeding [18]. The close connection between bone metabolism and eating is demonstrated by the association of nocturnal bone mineral loss with secretion of upper-intestinal hormone glucagon-like peptide-2 (GLP-2) within minutes of food intake, and by suppression of nocturnal bone mineral loss by subcutaneous injection of the hormone [19].

**Figure 3:**
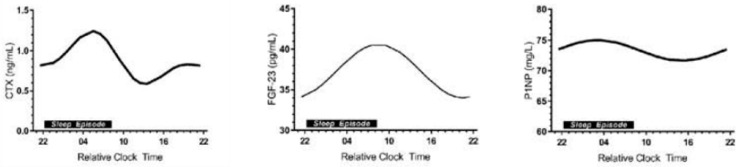
Circadian rhythms of three bone markers: CTX, marker of bone resorption, secreted by osteoclasts (**left** panel), FGF-23 (fibroblast growth factor-23) secreted by osteocytes (**center** panel), and P1NP or CICP, marker of bone formation, secreted by osteoblasts (**right** panel). Modified after [20], Reproduced with the permission of the publisher.

There was a similar lack of interest in the possible role of circadian timing of exercise in bone metabolism until the recent publication [20] documenting the circadian rhythms of CTX released by osteoclasts, of the fibroblast growth factor 23 from the mechano-sensing osteocytes, and of P1NP produced by osteoblasts (Figure 3).

In contrast to the recommendations of Turner and Robling providing exercise strategies to increase bone strength [12], none of which were validated in humans, recent research advances, and studies presented in this review, allow an updated and integrative report on how to effectively stimulate bone anabolism in postmenopausal women. The new evidence is that bone anabolism is remarkably responsive to chronological and circadian timing of meals and movement. The new findings show that (1) bone mineral resorption can be suppressed by higher movement momentum facilitated simply by increasing walking speed; that (2) an osteogenic outcome can be achieved by timing of meals before, rather than after, exercise, and by (3) a 40- to 45-min impulse, the necessary duration of effective mechanical stimulus; that (4) the anabolic response can double in size by insertion of a 7-h rest between two bouts of exercise; and that (5) circadian timing of exercise is important for stimulation of an anabolic response. In addition, bone sensitivity to exercise and nutrients depends on, and can be even potentiated by, their timing. 

## 2. Importance of Momentum and Power of Movement in Bone Anabolism

Momentum is the product of a mass and the velocity of its movement, and power is the work energy produced per unit time. Both concepts include an element of force, as in body resisting gravity or applying force during a task, but also, importantly, both concepts also include the element of movement timing. While these definitions are used in physics, and also are applied in sports to shorten running times or to produce greater distances in jumping or hurling objects, they have been less often recognized, or applied, in terms of bone anabolism. A well-known rare exception is the 30-year recognition of effectiveness of racket sports to increase BMC in the stroke arm [21].

Walking is a favorite human physical activity [22] and a good example of the general failure to apply the concept of momentum to this movement for the benefit of preservation of BMD in women. A systematic review and meta-analysis has labeled walking as an ineffective approach to protect BMD in perimenopausal and postmenopausal women [23]. However, by increasing its speed and momentum, walking can suppress bone mineral loss in healthy postmenopausal women [24].

In this study, 25 healthy postmenopausal women, motivated by a desire to lose weight, were recruited to walk 3 miles (4.8 km) per day in a shopping mall, either at the fastest possible speed (13 women), or at a comfortable, leisurely pace (12 women). Subjects were matched by age, body mass (76.8 kg in fast, and 78.3 kg in slow walkers), hormone-replacement status, and fitness level. Peak vertical forces at intensities corresponding to walking speeds were measured in nine women on a force plate in the laboratory. The supervised study lasted 15 weeks, and areal bone mineral density (aBMD), determined by DXA, was measured at the outset and after 15 weeks. 

**Figure 4:**
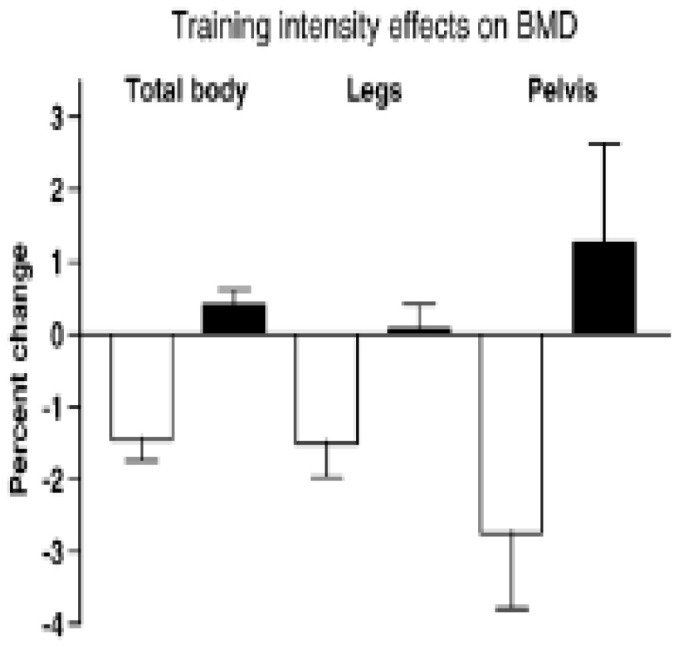
Percent change in BMD after 30 weeks of walking at two different velocities by healthy postmenopausal women. After [24] with the permission of the publisher.

The results showed that the average weekly participation was 4 days; walking intensity was 86% of maximal effort (VO_2_ max) at the fast walking speed of 6.3 km/h or 3.9 mph, and the distance of 5.4 km or 3 miles was covered in 42.5 min. The slow walkers exerted themselves at 47% of relative effort at the slow speed of 4.5 km/h or 2.8 mph, and the same distance was covered in 52.9 min. The aBMD of legs and whole body, but not of other sites, and lean mass of legs, but not of arms, was preserved or increased modestly after 15 weeks of high-velocity walking, compared to substantial losses for low-velocity walking (Figure 4). 

The conditions necessary for converting the ineffective mechanical stimulus of a comfortable walking speed of 5.4 km/h to an effective suppression of bone mineral loss in postmenopausal women simply involved raising the walking speed to 6.3 km/h, and thus increasing movement momentum. Increased momentum raised the physical effort above 74% of VO_2_ max and mechanical loading above 872.3 N or 1.22 multiples of body weight. Increased momentum neutralized the non-significant force differential in favor of fast walkers (900 vs. 850 N) at the same level of energy expenditure in both groups, which was unaffected by walking speed [25]. Higher momentum blocked BMD loss despite the shorter, 43-min completion time of a 3 mile distance, compared to the ineffective longer, 53-min course completion by the slow walkers. This study demonstrates that increasing momentum of movement, rather than differential mechanical loading, suppresses BMD loss in postmenopausal women. Therefore, these data provide a specific supplement to the general BHOF recommendation for weight-bearing and muscle-strengthening exercise for prevention of osteopenia, osteoporosis, and bone fractures.

## 3. Timing of Nutrient Intake before Exercise Is Necessary for Osteogenic Response in Postmenopausal Bones

**Figure 5:**
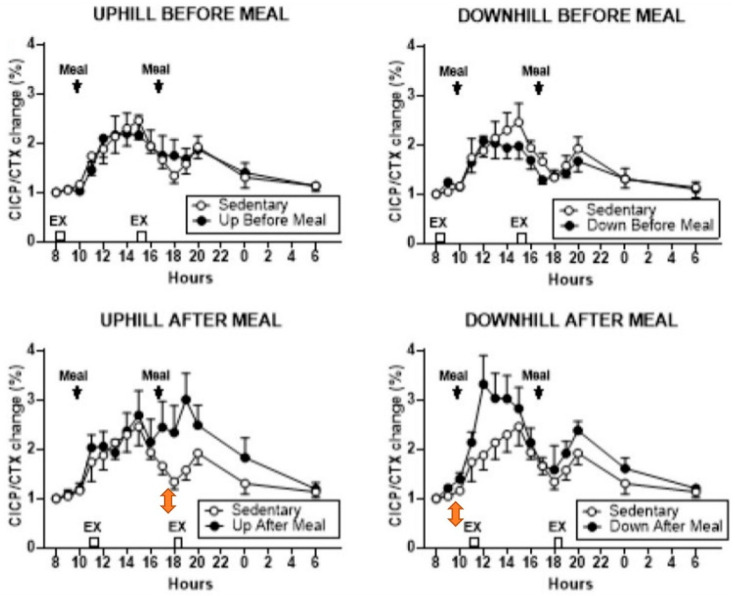
Percent change in the osteogenic serum CICP/CTX ratio between the sedentary trial and pre-meal exercise (**top** panels) and post-meal exercise (**lower** panels). Modified after [26], Reproduced with the permission of the publisher.

The purpose of study [26] was to examine the bone anabolic response to the timing of two meals relative to two exercise bouts separated by 7 h. Postmenopausal women with type 2 diabetes were selected for this study because the quality of their bones is diminished by insulin resistance which increases the risk of fractures despite their often normal or increased BMD [27]. In this study, 15 subjects, each, performed two of five trials: sedentary (SED), pre-meal and post-meal uphill (UBM and UAM), and downhill (DBM and DAM) exercise, respectively. Mechanical strain was manipulated through treadmill slope. Mechanical loading is reduced in uphill locomotion where increased muscle force attenuates gravity, and is increased in downhill locomotion where the two forces are additive [28]. This was made possible through a treadmill modification where a lever arm, powered by a mechanical jack, raised the rear end of the treadmill to a −6° slope relative to level surface, while uphill exercise operated on a +6° slope. Exercise bouts at 8 and 15 h preceded the daily weight-maintenance meals by 2 h, and exercise at 11 and 18 h followed the 10 and 17 h meals by 1 h. The meal macronutrient composition was 60% carbohydrate, 15% protein, and 25% fat. Specific foods were selected by Michigan Clinical Research Unit dieticians. The morning meal was an egg salad plate with multi grain bun, wheat roll with margarine, coleslaw, carrot sticks, skim milk and orange juice, graham crackers, and a serving of fresh fruit. The evening meal included a sandwich containing two slices of bacon, one slice of American cheese and 2 oz. of baked ham with green-leaf lettuce, wheat toast with diet mayonnaise, along with cooked broccoli, cauliflower, and carrots, plus tossed salad with diet French dressing, pretzels, 1.5 servings of fresh fruit, a carton of cranberry cocktail, and vanilla ice cream. The results showed that relative to the SED trial, the osteogenic response, defined as the ratio of osteogenic CICP marker over bone-resorptive CTX marker, increased in the first post-meal DAM (Figure 5, lower right panel), and in second post-meal UAM, exercise bouts (Figure 5, lower left panel). On the other hand, both UBM trials (Figure 5, upper panels) failed to affect the osteogenic ratio. 

**Figure 6:**
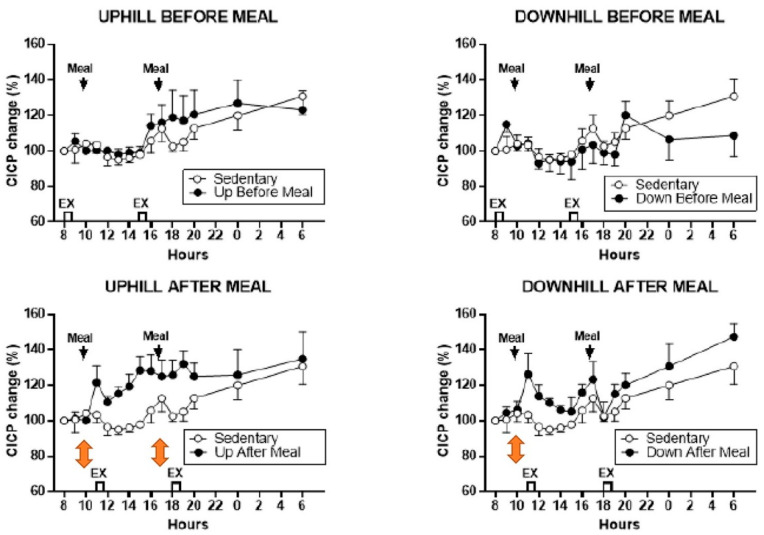
Percent changes in serum CICP between sedentary trial and exercise trials performed before meals (**top** panels) and after meals (**bottom** panels). Modified after [26], Reproduced with the permission of the publisher.

The osteogenic effect in this study was clearly achieved by increased secretion of CICP after post-meal exercise bouts and not by a change in CTX, which was unaffected under all exercise conditions. In the post-meal downhill trial, CICP concentration increased after the first meal and exercise bout (Figure 6, lower right panel). However, in the uphill post-meal trial, CICP concentration increased after both meal and exercise bouts (Figure 6, lower left panel), demonstrating that inserting a 7-h rest period between the two meals and exercise bouts doubles the anabolic effect in postmenopausal women as it did in the animal experiments (Figure 2B).

An osteogenic increase in the CICP/CTX ratio to post-meal exercise happened despite a 56% greater effort in UAM trials, and a 33.5% greater ground-reaction force in DAM trials caused by a difference in treadmill slope. Post-meal osteogenic response occurred at circadian times of 11 and 18 h in conjunction with 10 and 17 o’clock meals, in contrast to study [24] where the suppression of bone resorption occurred with exercise initiated at 8 h. Also, in study [26], pre-meal exercise, initiated at circadian times of 8 and 13 h, was not osteogenic. This further supports the conclusion that the timing of meals before the 8 (and 13) h exercise overrides the osteogenic circadian influence of post-meal exercise at these times. Dependence of increases in bone-formation marker CICP to post-meal, but not pre-meal, exercise adds another missing detail regarding the necessity of nutrient intake before exercise and the critical timing of meals and exercise to the general BHOF dietary recommendations. 

## 4. How Long Should an Exercise Bout Last to Produce an Anabolic Response in Post-Menopausal Bone? The Matter of Impulse Duration 

**Figure 7:**
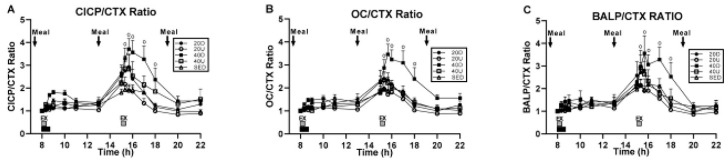
Changes in the osteogenic ratios between the three markers of bone formation CICP (**A**), osteocalcin, OC (**B**) and bone-specific alkaline phosphatase, BALP (**C**), and the marker of bone resorption CTX, to a single 40 min exercise at 8 am and to two 20-min bouts, both at 8 am and 3 pm in study [29], Reproduced with the permission of the publisher.

Study [29] was designed to find out whether the duration of effective exercise stimulus, or impulse, could be reduced by half. In medicine, impulse or force–time integral is applied for surgical treatment of atrial fibrillation [30]. Here, the impulse represents the necessary duration of electrocautery with current of sufficient voltage to remove a nerve triggering irregular heartbeat. In exercise, impulse is the area under each step’s force–time curve representing the sum of stepping force over time during the loading portion of the gait cycle. In two already mentioned studies, the osteogenic effect occurred with an exercise impulse of between 40 and 43 min. In study [24], suppression of BMD loss occurred after about 43 min of fast walking, and this successful precedent prompted the 40-min impulse assignment in studies [26,29]. Study [29] tested the hypothesis that a 20-min impulse will be as osteogenic as a 40-min one under the assumption that 40 min of exercise, especially in a protocol calling for two 40-min walking bouts, could be too challenging to some elderly women. The study, therefore, compared two 20-min exercise bouts spaced 7 h apart to a 40-min trial at 8 am, in addition to a SED control. As before, half of the 20-min and 40-min trials were uphill, and the other half downhill, to produce differences in mechanical loading. Forty healthy postmenopausal women, 8 each, were assigned to a no-exercise condition (SED) or to one of four exercise trials, 40 and 20 Up and 40 and 20 Down. Exercise was initiated at 8 am, one hour after eating a meal by all groups, and also 7 h later, two hours after the midday meal, by the 20-min groups. Three weight-maintenance meals of the same composition as in study [26] were provided at 7, 13, and 19 o’clock. Measurements were made of CICP, osteocalcin (OC), and bone-specific alkaline phosphatase (BALP), markers of bone formation, and of the bone-resorptive marker CTX. The osteogenic ratios CICP/CTX, OC/CTX, and BALP/CTX also were calculated. The results were that only the 40-min downhill exercise trial initiated at 8 o’clock increased the three osteogenic ratios CICP/CTX, OC/CTX, and BALP/CTX (Figure 7A, B, and C, respectively). None of the 20-min trials or the 40-min uphill trial produced an osteogenic response. The hypothesis was not confirmed, as the results demonstrated the necessity of a 40-min, and inadequacy of a 20-min, exercise impulse for stimulation of exercise-associated bone anabolism. 

Demonstrations of the necessary 40-min exercise impulse in studies [26,29] for stimulation of an osteogenic response, and of the 43-min impulse in study [24] for suppression of mineral loss in postmenopausal bone, provide a significant additional actionable detail to BHOF exercise prescription for prevention of osteoporosis and bone fractures.

## 5. Importance of a 7-h Rest Insertion between Two Exercise Bouts for Stimulation of Anabolism in Postmenopausal Bone

Studies [26,29] have, to the best of our knowledge, replicated for the first time in postmenopausal women the six-to-eight hour period of bone mechano-refractoriness first described in the animal studies [9,11]. Actual doubling of osteogenic CICP/CTX ratio and CICP concentrations was clearly observed in study [26] (Figure 6 and Figure 5, respectively). Four features, common to both of these rest-insertion studies were (1) implementation of two 40 [26] or one 40 and two 20 [29] minute impulses in exercise bouts separated by 7 h to examine the feasibility of doubling the anabolic effect; (2) application of two levels of mechanical loading through a modification of an ordinary treadmill for downhill, in addition to its usual uphill, operation [26]. (3) use of cellular markers of bone formation and resorption, and (4) utilization of Novel Pedar (Novel Electronics, St. Paul, MN, USA, https://www.novelusa.com/pedar, accessed on 10 February 2024) mechanosensitive shoe inserts and Novel Pedar software to quantify the mechanical loading generated by walking. 

The additive anabolic effect of two exercise bouts separated by seven mechano-refractory hours is seen most clearly in study [26] described previously in Section 3. There, both the 11 and 18 h post-meal uphill exercise, after the respective 10 and 17 h meals produced significant CICP increases (Figure 6, lower left panel), thus doubling the anabolic effect in the same trial. The finding that exercise-associated osteogenesis can be doubled in size by repeating it after a 7-h refractory period, is another important supplementary improvement to the general BHOF recommendation for prevention of osteopenia, osteoporosis and bone fractures. However, the differential increases in the osteogenic CICP/CTX ratio in uphill and downhill post-meal exercise in this study deserve additional investigation.

## 6. Importance of Appropriate Circadian Timing of Exercise for Optimal Anabolic Response of Postmenopausal Bone

**Figure 8:**
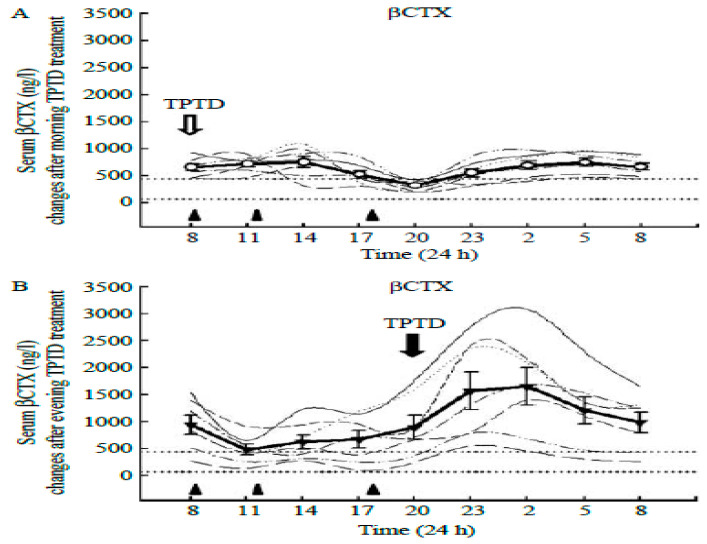
Effect of teriparatide (TPTD) administration to osteoporotic postmenopausal women at two circadian times, (**A**): 8 am, when the nocturnal CTX peak is suppressed, and 8 pm (**B**), when CTX concentration is not suppressed. Reproduced from [33] with permission of the publisher.

Exercise starting times in our three studies [24,26,29] were designed without consideration of possible circadian effects. However, a possible circadian explanation of the unexpected data regarding 40-min post-meal uphill exercise in study [29] became of interest after documentation of circadian rhythms of bone markers in bone health [20]. This report documented the circadian rhythms (Figure 3) in the resorption marker CTX released by osteoclasts (left panel), in the FGF-23 produced by strain-detecting osteocytes (center panel), and in bone formation marker P1NP (right panel). We examined the possible role for bone marker or hormonal circadian rhythms in the discrepant osteogenic outcomes between the 40-min uphill and downhill trials in study [29]. A possible clue for the absence of the anabolic effect after 40-min uphill exercise was in the well-known opposite effects of parathyroid hormone (PTH) on bone metabolism depending on the pattern of its secretion. When plasma calcium declines, usually due to inadequate calcium intake or to increased urinary calcium loss, secretion of PTH rises in a sustained fashion, which is clinically recognized as secondary hyperparathyroidism [16], and causes bone resorption. This corrects circulating calcium deficiency as PTH-induced calcium release resorbs bone mineral from the vast mineral reserve of the human skeleton (1 to 2 percent of body weight). On the other hand, when PTH is released intermittently in a pulsatile fashion, as is the case in exercise [31], it actually stimulates bone mineral formation [32]. This well-known reversal of the sustained resorptive PTH action to bone mineral accretion with pulsatile PTH secretion is used in medicine with injections of synthetic PTH analog, teriparatide (TPTD), to treat osteoporosis [33], Application of TPTD is a circadian event, because a circadian 8 h admnistration to osteoporotic postmenopausal women completely abolished nocturnal circadian CTX rhythm [33] (Figure 8A), while a 20 h injection did not (Figure 8B). In a several-month-long follow-up study [34], BMD in women with postmenopausal osteoporosis increased to a greater extent with 8 h TPTD injections than when the TPTD was administered at 20 h. Initiating exercise at 8 am circadian time also suppressed bone mineral loss after 15 weeks of walking in study [24].

Exercise at 8 h coincides with the decline in bone resorption marker CTX towards its nadir at mid-day (Figure 3, left panel, Figure 8B), when the FGF-23, osteocyte mechanosensitive signal [20] is close to its 9 h acrophase (Figure 3, center panel).

**Figure 9:**
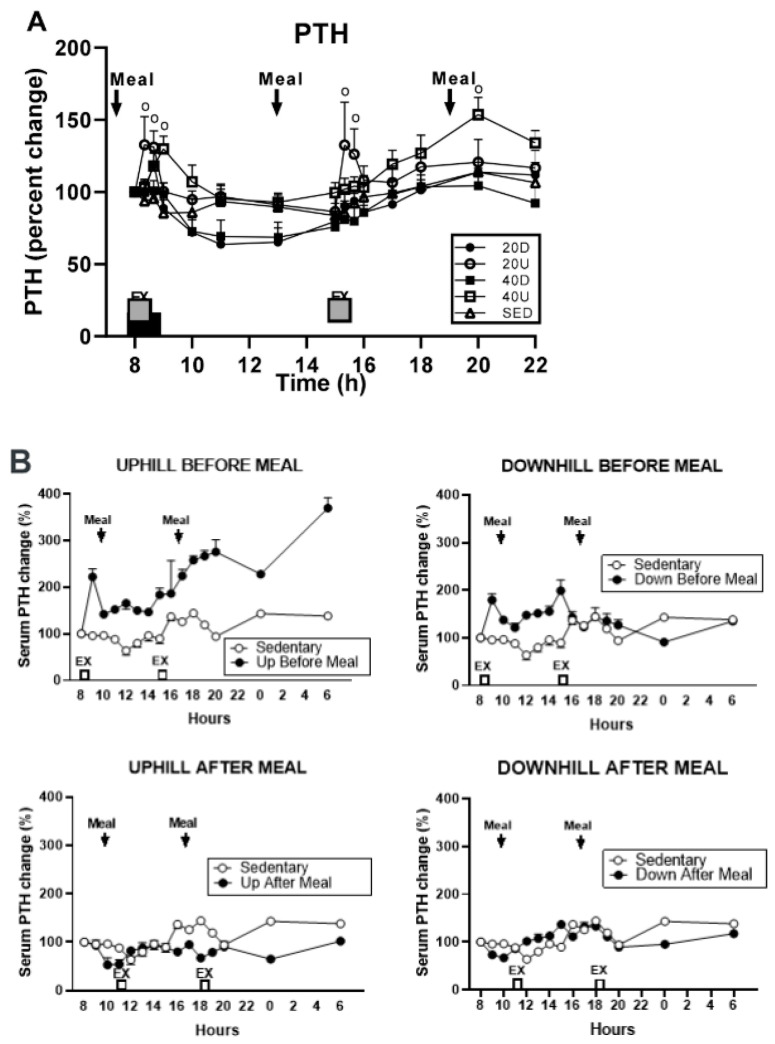
Percent changes in plasma parathyroid hormone (PTH) in response to 40 min exercise. (**A**): Comparison of PTH responses to either a 40-min exercise at 8 h or 20-min exercise bouts at 8 and 15 h [27]. (**B**): Percent changes in plasma parathyroid hormone (PTH) comparing 40-min pre-meal exercise bouts at 8 and 15 h to 40-min post-meal bouts at 13 and 18 h. A control sedentary condition was included in [26], Reproduced with the permission of the publisher.

The evidence that bone resorption can be induced by sustained oversecretion of PTH [16] and converted to mineral accretion with solitary pulses of PTH [32] or injections of TPTD [33] prompted us to reexamine the pattern of PTH secretion in study [29], where 40-min uphill exercise initiated at 8 h failed to produce elevation of CICP/CTX, OC/CTX, and BALP/CTX ratios but downhill exercise did (Figure 7). PTH was also measured in study [26], where the 40-min uphill exercise initiated at circadian time 8h was not osteogenic, as it was performed before eating. PTH concentrations after 40-min uphill exercise in both of these studies show opposing patterns of this hormone’s secretion when exercise is initiated at 8 h. In study [29], the osteogenic downhill exercise elicited a distinct PTH peak after the onset of exercise, with a decline in PTH concentration throughout the rest of the trial (Figure 9A). The concurrent uphill exercise elicited a PTH pulse, followed by a sustained elevation of the hormone concentration to the end of the trial. After the first pre-meal uphill exercise in study [26], PTH concentration increased dramatically over 22 h (Figure 9B, top left panel) suggesting the operation of its bone-resorptive action [16]. In the concurrent downhill pre-meal trial, PTH concentration was moderately increased only between the two exercise bouts (Figure 9B top right panel). 

The consistent resorptive pattern of PTH secretion in both studies [24,29] in response to the uphill exercise at their circadian 8 h onset, and the osteogenic effect of PTH pulses of downhill exercise in these studies, indicate important circadian interaction between the 8 h circadian timing of initiation of exercise, different mechanical loading due to treadmill slopes, and differential PTH actions on CTX effects on bone metabolism based on the pattern of its release. Therefore, this circadian effect of exercise initiated at 8 h on bone metabolism needs to be added to BHOF general exercise recommendations for prevention of osteoporosis and bone fractures.

Additional information of interest regarding circadian effects on bone anabolism was obtained in post-meal trials of study [26]. Here, 40-min exercise initiated at 11 and 18 h did not appear to change CTX concentration, but, instead, increased CICP concentration (Figure 6) and osteogenic CICP/CTX ratios (Figure 5). In this study, exercise initiated at circadian 8 and 15 h had no osteogenic effects (Figure 5 and Figure 6, upper panels), presumably because exercise was performed in the fasted state. How and why the absence of nutrient intake blocks osteogenic effects during pre-meal exercise initiated at the circadian times 8 and 15 h requires additional investigation. 

## 7. Can Timing of Nutrients and Exercise Increase Bone Sensitivity and Allow Anabolic Responses to a Second Subthreshold Mechanical Stimulus and in Its Absence? 

The prevailing view in the field of bone research is that a substantial supra-threshold mechanical stimulus is a necessary condition for any anabolic bone remodeling [35,36]. Yet in several protocols that manipulate the timing of nutrients and exercise, anabolic changes in bone can be triggered at sub-threshold mechanical loading either by a meal in the absence of the second mechanical stimulus [27], or after delayed access to food [37], and by timed blood-flow restriction (BFR) [38]. 

Anabolic bone response was documented after a weight-maintenance meal in the absence of an exercise bout, seven hours after the first downhill exercise bout initiated at circadian time 8 h in the in study [29] (Figure 7). This osteogenic response started two hours after the 13 h mid-day meal and took place between the circadian times of 15 and 20 h. This response coincided with the onset of the second exercise bout in the four non-osteogenic 20-min groups. The coincidence of the onset of this osteogenic response after a meal in the absence of exercise, at the end of a 7 h mechano-refractory period, suggested the existence of a 7 h nutrient-sensitive refractory period in parallel to the 7-h period of mechano-refractoriness. It appears that after a 7 to 8 h of refractoriness, bone regains sensitivity both to mechanical stimulation and to nutrients. This inference represents a hypothesis that requires additional research, but also opens a new perspective on the interaction between concurrent variations in osteogenic bone sensitivity to nutritional and mechanical stimulation.

**Figure 10:**
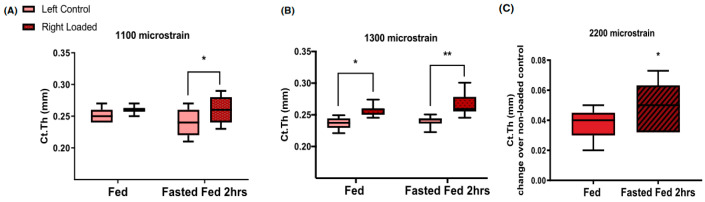
Increases in the thickness of young mouse tibia cortical bone under two suprathreshold loading protocols at 1300 (**B**) and 2200 (**C**) microstrain, and under subthreshold 1100 microstrain (**A**) loading during normal ad libitum feeding (fed) and after 2 h of refeeding in response to a 16-h fast (Fasted Fed 2 h). * *p* < 0.05, ** *p* < 0.01. Modified after [37], Reproduced with the permission of the publisher.

The capacity of bone to respond to subthreshold mechanical stimulation was also demonstrated after substantial nutrient deprivation. In study [37], tibiae of 17-week old mice received 40 cycles of loading, ranging between 1100 and 2200 microstrain in the type of apparatus shown in Figure 1A. The anabolic bone response was examined after mechanical stimulation during two hours of refeeding that followed a 16-h fast. Refeeding after the 16-h fast approximately doubled the thickness of the tibial cortex at supra-threshold mechanical loads of 1300 and 2200 microstrain (Figure 10B and Figure 10C, respectively). It also doubled the thickness of the tibia cortical bone at the subthreshold 1100 microstrain load (Figure 10A), the mechanical loading which was ineffective during subthreshold mechanical loading under ad-libitum feeding conditions. This animal model of nutritional bone sensitization could theoretically increase osteogenesis in humans by applying exercise during the refeeding day after a day of fasting.

The data in these two instances reveal a knowledge gap about the capacity of bone to change its sensitivity to mechanical stimulation in response to changes in its pre-exercise access to food. Both approaches provide the opportunity to study how changes in bone access to nutrients change its exercise-associated anabolic responsiveness.

A final illustration of the ability of bone to generate an osteogenic response at subthreshold mechanical loads features application of a blood-flow restriction (BFR) procedure during exercise training [38]. The BFR or KAATSU practice was invented in Japan, but is now more widely practiced in both joint-reaction [39], and ground-reaction [40] training. The practice entails application of tourniquets at the base of limbs combined with intermittent exercise training that produces intermittent BFR. While BFR is not equivalent to manipulation of bone access to nutrients, circulation provides the bone with nutrients, hormones, and oxygen. During muscle contractions, blood flow and access to these needed substances is intermittently reduced. The main attraction of BFR is that muscle hypertrophy, and some increases in BMC, can be achieved at sub-threshold resistance loads [38]. The practice attracts not only young and healthy individuals who desire to produce maximal muscle hypertrophy, but also older or disabled individuals who cannot, or do not wish to, engage in heavy-loading exercise. The procedure for the latter population is not without the risk of clotting and blood cell damage, as a small number of cases of pulmonary embolism and rhabdomyolysis attest. 

These three conditions suggest that a supra-threshold mechanical loading is not a prerequisite for anabolic bone response under all circumstances. The mechanism of bone remodeling after different types of challenges appears to be a response to a periodic interference with its nutrient or blood oxygen supply in the context of intermittent mechanical loading. Further molecular explanation of the mechanism of these phenomena requires additional research attention.

## 8. Limitations of the Review

The purpose of this review was to bring together studies that explain the relevance and importance of timing of exercise and nutrients in suppression of bone mineral loss and in facilitation of anabolic responses in postmenopausal bone. As such, the review covered wide-ranging issues, including discussion of the roles of both chronological and circadian timing of exercise and meals and of variables contributing to the efficacy of osteogenic stimulus and to the mechanical and nutritional refractoriness. The diversity of these issues required selective rather than comprehensive archival documentation of key points. Usually, the key early reports of significant facts in the impact of mechanical loading on bone biology are cited, leaving out many alternate, albeit deserving, papers. 

The five main points on the importance of timing of exercise and meals in anabolic remodeling of postmenopausal bone being made in this review are, (1) the relative importance of momentum and power as opposed to any level of mechanical loading per se, (2) the necessity of eating a substantial meal before an exercise bout, (3) the importance of the duration of an effective mechanical stimulus or impulse, (4) the importance of 6- to 8-h spacing between the exercise bouts to double the anabolic effect, and (5) significant dependence of the anabolic outcome on the circadian timing of exercise. They are based on a relatively small number of studies. Only two human studies that included a 7-h rest between two exercise bouts in human subjects, [26,27], are presented and, to the best of our knowledge, currently exist. The timing of meals and exercise in study [26] engaged diabetic rather than healthy postmenopausal women, so a generalization of these results will require additional research with healthy subjects. Since study [29] produced unusual results that were not specifically manipulated by the study design, discussion about the relevance of circadian timing of exercise in anabolic bone responses had to rely on data from studies by other authors [33,34]. It will, therefore, be necessary to validate these conclusions by additional research that directly manipulates relevant variables.

Despite the above-mentioned limitations, this review attempts to fill in the gaps in the prevailing appropriate, but very general and non-specific, recommendations by BHOF on how to use exercise and nutrition to reduce bone mineral loss and bone fractures in osteopenia and osteoporosis. The following recommendations provide suggestions on how to effectively utilize responsiveness of bone to timing of exercise and nutrition to obtain desired anabolic effects. Besides providing additional specific details, the review and recommendations highlight several knowledge gaps in our understanding of how bone recognizes an effective anabolic stimulus and how it orchestrates the 6- to 8-h period of mechano-refractoriness and nutritional refractoriness.

## 9. Discussion and Action Recommendations

The specific recommendations in this review can assist those who need to reduce the risk of osteoporosis and bone fractures as well as encourage those who can perform this line of research to provide scientific answers to the outstanding questions. A summary of main points is diagrammed in Table 1, where it may serve as a quick guide to the selection of appropriate action.

## 10. Recommendations

The following recommendations are based on the results obtained in studies with postmenopausal women weighing about 78 kg or 172 lbs. Hence, effects of recommendations on manipulation of variables that affect force per unit time are likely to be affected by the body mass of subjects following these recommendations and may accordingly need to be modified. Also, results obtained from treadmill data where the slopes affected the magnitude of mechanical loading are here assumed to work in subjects who are likely to be walking on level ground. Both assumptions require experimental evaluation.

It also is important to consider that these recommendations pertain specifically to improving bone health and not to overall health benefits of exercise. The 2011 consensus of the American College of Sports Medicine [41] has defined desirable volumes of exercise for optimal health as 150 or more minutes per week of moderate intensity, and 75 weekly or more minutes at vigorous pace. The American Heart Association [42] and BHOF [1] have adopted these ACSM recommendations for the volumes and intensity of exercise as also pertaining to optimal heart and bone health, respectively. Fast walkers in study [24] generated 170 min of vigorous exercise per week, and slow walkers, 211 min per week. Therefore, this study protocol more than doubled the recommended volume of vigorous exercise and produced 40% higher volume of recommended moderate-intensity exercise. Walking at higher velocity and momentum specifically benefitted bone mineral, but the walking volumes achieved at both speeds amply benefitted overall and cardiovascular health. An added benefit of walking even at a comfortable pace for 15 weeks is that the same subjects in the study [24] lost more body fat than the fast walkers [43].

Recommendation 1A: **Select 8 h circadian time for suppression of bone mineral loss with exercise.**
  For suppression of bone mineral loss, postmenopausal women are advised to engage in ground-level exercise covering 3 miles (4.8 km) in 40 to 43 min at a pace of 6.3 km/h (3.9 mph) at 8 h circadian time, a practice which suppressed bone mineral loss after exercising 4 days a week for 15 weeks and suppressed CTX, but not when performed on uphill exercise slope. Exercise should be performed one to two hours after a weight-maintenance meal. The rationale for each statement is explained in Section 2, Section 6, Section 3 and Section 4, respectively.
Recommendation 1B: **Select 11 and/or 18 h circadian time for stimulation of bone-formation marker CICP with exercise.**
  To increase the concentration of bone formation marker CICP, engage in 40 to 45 min of exercise starting at 11 h (and 18 h if exercising twice a day) covering 3 miles (4.8 km) at a pace of 6.3 km/h (3.9 mph). Exercise should be performed one to two hours after a weight-maintenance meal. The rationale for each statement is explained in Section 3, Section 4 and Section 6, respectively.

These two recommendations about the specific circadian timing of exercise entail several uncertainties despite the phenomenological demonstration of their osteogenic effectiveness. First, it is not clear why starting exercise at 8 h, close to the nadir of CTX rhythm ([20,33]) should suppress the expression of this marker’s circadian rhythm. The data would suggest that exercise at this circadian time takes about 12 h to exert its effect. Second, it is not clear why exercise initiated at 8 h [24], or PTH injection at that time, suppresses bone mineral loss and expression of CTX rhythm [33], while exercise initiated at 11 and 18 h stimulates increased release of CICP, but has no effect on CTX concentration [26]. It should be investigated whether a slight difference in timing of the circadian rhythms of individual bone markers ([20], Figure 3) may be the cause. Third, it is not clear why the osteogenic effect of circadian timing of exercise at 8 h is cancelled when a meal is not eaten prior to starting exercise [26] (Figure 5 and Figure 6, upper panels). More research toward answering these questions will expand our understanding of the interactions between circadian timing of exercise, nutrition, and bone marker responses.

Recommendation 2: **Increase the speed of exercise to achieve a higher momentum.**
  To convert ineffective ground-level walking at the moderate speed of 5.4 km/h over a 3-mile (5.4 km) course into exercise that suppresses bone mineral loss, postmenopausal women should walk faster than 6.3 km/h or 3.9 mph for 43 to 45 min. Another prerequisite to make this practice osteogenic is to eat a weight-maintenance meal 1 to 2 h before exercise. Finally, exercise should be initiated at the circadian times of 8 (and 15) h or 11 (and 18) h, if two exercise bouts are involved. The rationale for each statement is explained in Section 2, Section 3, Section 4 and Section 6, respectively.
Recommendation 3: **Eat a weight-maintenance meal before the exercise bout.**
  Eating of a weight-maintenance meal one to two hours before exercise appears to be a requirement for elicitation of an anabolic response. Pre-meal exercise appeared to cancel osteogenic effectiveness of the 8 h circadian start of exercise. The prerequisites of faster walking momentum and 43- to 45-min exercise impulse also apply. The rationale for each statement is explained in Section 3, Section 6, Section 2 and Section 4, respectively.

Study [26] provided a much needed examination of the role of timing of meals and exercise in the responsiveness of bone to exercise, a relationship that has not been often pursued in loading-focused bone research. The dependence of anabolic outcome on post-meal exercise [26] confirmed the importance of meal eating and of meal-associated release of gut hormone GLP-2 [18] in diurnal mineral accretion and suppression of nocturnal mineral loss associated with the release of bone resorption marker CTX [19]. However, the clear demonstration of the necessity to eat a weight-maintenance meal before exercise to elicit an osteogenic response [26] was performed in type-2 diabetic postmenopausal women. Because their diabetic state could have affected the response, it is necessary to replicate this study with healthy postmenopausal women.

Recommendation 4: **Sustain a walking impulse for 40 to 43 min.**
  It is necessary to sustain the duration of ground-level walking for 43 to 45 min. Reduction of this impulse to 20 min per exercise bout has no osteogenic effect. Other prerequisites such as necessary walking speed, a pre-exercise meal, and specific circadian timing also apply. The rationale for each statement is explained in Section 4, Section 2, Section 3 and Section 6, respectively.

The prerequisite of a 40-min exercise bout or impulse to produce osteogenesis in studies [26,29] addresses the unresolved question of how bone determines efficacy of an osteogenic mechanical impulse and initiates a subsequent period of refractoriness to mechanical stimulation. Recognition of an effective duration of mechanical stimulus or impulse mandates insertion of rest periods in order to overcome bone mechano-refractoriness and regain its sensitivity to additional mechanical stimulation. Several early animal studies, using the technique illustrated in Figure 1, have shown that distributing the same amount of mechanical loading during the day produces greater bone mineral accrual [44] and bone strength [45] and shortens the refractory periods after such shortened loading bouts separated by inserted rest period compared to delivering the same load all at once in a 360 loading cycle within 3 min. A detailed analysis [46] of the effect of distributing the same loading energy (1300 to 2300 microstrain at a frequency of 2 Hz) tested the osteogenic effects of two 2-min (90 s) bouts separated by 6 h, or four 45-s bouts, or six 30-s bouts, separated by 3 and 2 h over a period of 9 and 14 h, respectively. They found that breaking down the same mechanical load into 6 episodes, increased endosteal lamellar bone formation rate (ELBFR) tenfold in the loaded relative to unloaded bone. Breaking the same mechanical loading into 4 episodes increased ELBFR 8-fold, but breaking it into 2 episodes produced fourfold the same as performing a single load, that is ELBFR increased it only fourfold, the same as with a single loading. Reasoning by analogy to the study [46], it may be worth checking out whether carrying out four walking bouts with 10-min impulses, each bout separated by a 3-h rest over a period of 9 h, would produce a greater anabolic effect compared to a single exercise bout that was assigned in our studies a 40-min impulse. Therefore, additional studies should determine whether the prerequisite for a 40- to 45-min impulse for osteogenesis during high-momentum walking in postmenopausal women [24,26,29] is an artifact produced by the absence of intermittent rest periods within the required 40 impulse. A possible influence of the circadian time should also be checked as the single loading cycle was performed at 11 h, and 90, 45, and 30 s loading episodes at 9 and 15 h, 9 through 17 h, and 8 through 8 h, respectively.

Recommendation 5: **Repeat effective exercise stimulus at 18 h after the first one initiated at 11 h to double the anabolic effect.**
  To double the release of bone-formation marker CICP, eat a weight maintenance meal at 10 and 17 h before initiating two bouts of exercise at 11 and 18 h; walk for 40 to 45 min at the necessary speed of 6.3 km/h (3.8 mph) during both exercise bouts. A corresponding study with two exercise bouts initiated at 8 h and 15 h under the same conditions has not been conducted. The rationale for each statement is explained in Section 3, Section 5 and Section 2, respectively.

Studies with mechanical loading of animal limb bones found that inserting a rest period between two effective bouts of mechanical loading overcame a period of mechano-refractoriness and restored bone sensitivity for mechanical stimulation [11]. Repeating a second effective exercise bout after the 6 to 8 h period of mechano-refractoriness triggered by the first exercise bout, allowed a doubling of the anabolic effect (Figure 2B). Studies [26,29] were apparently the first attempts to examine this phenomenon in humans, but only study [26] succeeded in demonstrating doubling of circulating CICP after both meals and exercise bouts in postmenopausal women because it contained all of the necessary preconditions of appropriate walking speed and momentum, a 40-min impulse, and 7 h recovery period between two identical exercise bouts (Figure 5 and Figure 6). Since in study [29], the necessary 40-min impulse was not repeated twice with insertion of a 7-h rest period, and the 20-min impulse was not sufficient to elicit an anabolic bone response, doubling of the anabolic response to exercise at the 8 h circadian period has not been accomplished.

Recommendation 6A: **Consider eliciting anabolic bone responses in the absence of second bout of mechanical stimulation.**
  If physical constraints or a reduced level of fitness preclude exercising at recommended intensities and durations twice a day, consider increasing the osteogenic ratios of CICP/CTX, OC/CTX, and BALP/CTX after only one 8 h bout walking on level ground but not with an uphill treadmill slope. Eat a weight-maintenance meal at 7 h before walking for 40 min at 6.3 km/h (3.9 mph). Increased osteogenic response will be triggered by the 13 h weight-maintenance meal in the absence of a need for a second exercise bout. The rationale for each statement is explained in Section 1, Section 7, Section 3, Section 2 and Section 5, respectively.

The observation in study [29] that a 40 min exercise bout of appropriate speed initiated at 8 h elicited increased osteogenic ratios of CICP/CTX. OC/CTX, and BALP/CTX after a weight-maintenance meal at 13 h revealed that there may be parallel periods of bone sensitivity to mechanical and nutritional stimulation that are restored after 6 to 8 h of refractoriness.

Recommendation 6B: **Consider eliciting anabolic bone responses during refeeding after an extended fast with a subthreshold mechanical stimulus.**
  An animal study demonstrated that bone sensitivity to nutrients can be increased during exercise that follows post-fast refeeding. Although this has not been tried in humans, the example of fasting-induced increase in bone sensitivity to nutrients may be appropriate for individuals who practice intermittent fasting. It would be worth finding out whether reduced momentum and impulse of exercise would produce enhanced osteogenic effects if such exercise were initiated within an hour of ad libitum re-alimentation. The rationale for each statement is explained in Section 7, Section 2 and Section 4, respectively.
Recommendation 6C: **Consider eliciting anabolic bone responses during subthreshold exercise under blood-flow restriction.**
  Blood-flow restriction (BFR) or KAATSU is practiced by individuals who seek to increase muscle mass and strength [39,40] by training at subthreshold training effort. In addition to facilitating hypertrophy, this practice has also been found to increase BMD [38]. The practice has spread from Japan across the globe, but is not without some clotting risks.

This list of recommendations provides a counterpoint to reviews that simply list conditions and circumstances that affect postmenopausal bone biology [47]. Instead of just listing such factors, this set of submitted actionable recommendations provides more specific recent evidence-based details on how to effectively utilize the chronological and circadian timing of exercise and nutrients in the prevention of osteoporosis, bone weakness, and fractures. The review also identifies knowledge gaps in bone responses to timing of meals and exercise and encourages research toward a better understanding of how bone detects adequacy of a mechanical stimulus and determines duration of post-exercise rest necessary to restore its sensitivity to mechanical stimulation and nutrients.

## Figures and Tables

**Table 1:** Recommendations for effective utilization of bone sensitivity to timing of exercise and nutrients for prevention of osteoporosis.

1. Select appropriate circadian time											
Symbol	Key Variable	Necessary Conditions	Reference	Figure	Effective?	Symbol	Key Variable	Necessary Conditions	Reference	Figure	Effective?
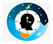	8 h	EAM IMP = 43 SP = 6.3 (3.9)	[24]	4	**YES**	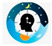	8 h 20 h	EBM DT, UT EAM UT IMP = 53, SP = 4.5	[24] [29]	4	**NO** **NO**
	8 h	TPTD #	[33] #	9A #	**YES**		20 h	TPTD #	[33] #	9B #	**NO**
	11 h 18 h	2 EBs, EAM IMP = 40, UT, DT	[26]	5 lLP 5 LP	**YES**		8 h 15 h	2 EBs, EBM, IMP = 40, UT, DT	[26]	5 lUP 5 rUP	**NO**
**2. Increase speed and momentum of exercise**											
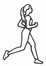	SP > 6.3 (3.9)	ST = 8 h, EAM DS = 4.8 (3)	[24]	4	**YES**	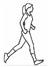	SP < 4.3 (2.7)	ST = 8 h, EAM DS = 4.8 (3)	[24]	4	**NO**
**3.** **Eat a weight-maintenance meal before the exercise bout**											
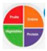	EAM	2 EBs, IMP = 40 ES = 11, 18	[26]	5 LP 6 LP	**YES**	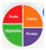	EBM	2 EBS, IMP = 40 ES = 8, 13	[26]	5 UP 6 UP	**NO**
**4.** **Sustain the walking impulse for 40 to 43 min**											
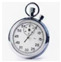	IMP = 43	LS, EAM, SP > 6.3 (3.9), DS = 4.8 (3)	[24]	4	**YES**	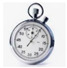	IMP = 53	LS, EAM, SP < 4.5 (2.8), DS = 4.8 (3)	[24]	4	**NO**
	IMP = 40	EAM, DT, ST = 8	[27]	7	**YES**		IMP = 20	EAM, DT, ST = 8	[27]	7	**NO**
**5.** **Repeat effective exercise stimulus after 7 to 8 h for a double benefit**											
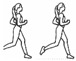	RI = 7 2 EBs	EAM, DT, UT ST = 13, 18	[26]	5 LPs 6 LPs	**YES**	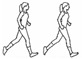	RI = 7 2 EBs	EBM, DT, UT ST = 8, 15	[26]	5 UPs 6 UPs	**NO**
**6. Opt for osteogenic benefit with subthreshold exercise intensity**											
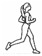	RI = 7 1 EB	ST = 8, EAM IMP = 40	[27]	7	**YES**	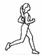	RI = 7 2 EBs	ST = 8, 15, EAM IMP20	[27]	7	**NO**
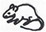	RI = 12 1 EB	PRF	[37] *	9	**YES**		RI = 0, AL	AL	[37] *	9	**NO**
	BFR		[38]		**YES**		No BFR		[38]		**NO**

Table legend: AL = ad libitum feeding in mice; BFR = blood-flow restriction; DS = necessary distance to be walked in km (miles); DT = downhill treadmill exercise at −6°; IMP = impulse or duration of effective exercise in minutes of walking, EAM = exercise 1 to 2 h after eating a substantial meal; EBM = exercise 1 h before a substantial meal; EB = exercise bout(s); l = left; LP = Lower panel(s); LS = walking on level surface; PRF = post-refeeding in the mouse study (not done in humans) r = right; RI = Insertion of rest in chronological hours; SP = speed of effective exercise in km/h (miles/h); ST = exercise starting time in military time; TPTD = teriparatide, synthetic parathyroid hormone; UP = upper panel(s); UT = uphill treadmill at +6°; # teriparatide (TPTD) injection to postmenopausal women with osteoporosis; * mouse study [37], not tried in humans.
